# More Than Skin Deep: Loculated Pericardial Effusion Causing Localized Cardiac Tamponade Due to Cutibacterium acnes Pericarditis

**DOI:** 10.7759/cureus.116232

**Published:** 2026-09-14

**Authors:** Samuel Daneshvar, Justin Sharim, Asim M Rafique, Rebecca Y Linfield, Ramin Tabibiazar

**Affiliations:** 1 Cardiology, University of California Los Angeles David Geffen School of Medicine, Los Angeles, USA; 2 Infectious Disease, University of California Los Angeles David Geffen School of Medicine, Los Angeles, USA

**Keywords:** cutibacterium acnes, infective pericarditis, loculated pericardial effusion, pericardial adhesion, regional tamponade

## Abstract

Acute pericarditis, or inflammation of the pericardial sac, is a common cause of chest pain with a broad range of etiologies, including viral, bacterial, traumatic, rheumatologic, and malignant causes; however, most cases are idiopathic. Acute pericarditis is associated with the development of a pericardial effusion in many patients. In rare cases, the effusion may progress to cardiac tamponade, a life-threatening condition in which increased intrapericardial pressure impairs cardiac filling through external compression of the cardiac chambers. Pericardial effusions are typically circumferential, and tamponade is classically recognized by compression of the lower-pressure right-sided cardiac chambers. However, atypical presentations can occur in which the left-sided chambers are primarily affected. Prompt recognition of this condition is essential to facilitate urgent or emergent pericardiocentesis.

We present the case of a 73-year-old man with a history of coronary artery bypass grafting (CABG) who developed a loculated pericardial effusion causing localized cardiac tamponade, necessitating urgent pericardiocentesis. Subsequent pericardial fluid cultures grew *Cutibacterium acnes (C. acnes)*, a common skin colonizer that was considered the causative organism of infective pericarditis based on the patient's clinical presentation. This report highlights the atypical echocardiographic findings of a loculated pericardial effusion causing localized cardiac tamponade. It also highlights the diagnostic difficulty in determining whether *C. acnes* is a pathogenic organism.

## Introduction

The pericardium is a fibrous sac that surrounds the heart. It is composed of two layers: a visceral layer adherent to the cardiac epicardium, which reflects onto the thicker parietal layer that adheres to the central tendon of the diaphragm, the posterior sternum, and the roots of the great vessels [1]. Typically, 20-60 mL of pericardial fluid is present between the layers of the pericardium. Acute pericarditis, or inflammation of the pericardial sac, has a broad differential diagnosis, including infectious, inflammatory, traumatic, and malignant etiologies, although most cases remain idiopathic.

In approximately 50-65% of cases, acute pericarditis leads to the abnormal accumulation of pericardial fluid, known as a pericardial effusion [2]. While acute pericarditis typically leads to small pericardial effusions, in rare instances (approximately 3% of cases), these effusions can be hemodynamically significant, leading to cardiac tamponade, a condition in which the pericardial effusion impairs cardiac filling [3]. If left untreated, cardiac tamponade can be fatal. Pericardial effusions tend to be free-flowing within the pericardial potential space and circumferential, typically first affecting the lower-pressure right-sided cardiac chambers. However, adhesions within the pericardial space can produce loculated pericardial effusions, potentially leading to regional cardiac tamponade.

In this report, we describe the case of a 73-year-old man with a history of coronary artery bypass grafting (CABG) who presented with a loculated pericardial effusion causing tamponade. Subsequent fluid cultures grew *Cutibacterium acnes (C. acnes)*, leading to the presumed diagnosis of acute infectious pericarditis. Prompt identification of infectious pericarditis and cardiac tamponade is essential, as early drainage and targeted antimicrobial therapy are critical to maintaining hemodynamic stability and preventing further complications.

## Case presentation

A 73-year-old male with a prior medical history of coronary artery disease (CAD) with CABG performed four years previously, as well as mitral valve prolapse with associated mild mitral regurgitation, presented to the emergency room with a two-week history of progressive dyspnea. He had initially noted these symptoms with light jogging, which progressed to the point that he had shortness of breath with walking. He presented to the emergency room when his shortness of breath became associated with pain in the bilateral lower jaw and bilateral shoulders. He denied chest pain; however, he noted that he did not have chest pain when he was diagnosed with coronary artery disease. He denied having any fevers before presenting to the emergency room.

Regarding the patient's CAD, it was initially identified on a screening CT performed to assess coronary artery calcification. Subsequent stress testing showed diffuse ischemia, with a coronary angiogram demonstrating severe multivessel disease, for which he underwent an uncomplicated CABG four years prior. In the emergency room, his initial vitals were as follows: pulse 80, BP 93/66, respiratory rate 18, oxygen saturation 95%, and temperature 36.6°C. ECG demonstrated normal sinus rhythm with low voltage in the precordial leads and nonspecific ST-T wave changes, similar to prior ECGs obtained after CABG (Figure 1). Chest X-ray demonstrated a normal mediastinal silhouette with pulmonary venous congestion (Figure 2). Initial high-sensitivity troponin I was 35 ng/L (upper limit of normal 5 ng/L), with subsequent levels increasing to a peak of 174 ng/L before downtrending.

**Figure 1:**
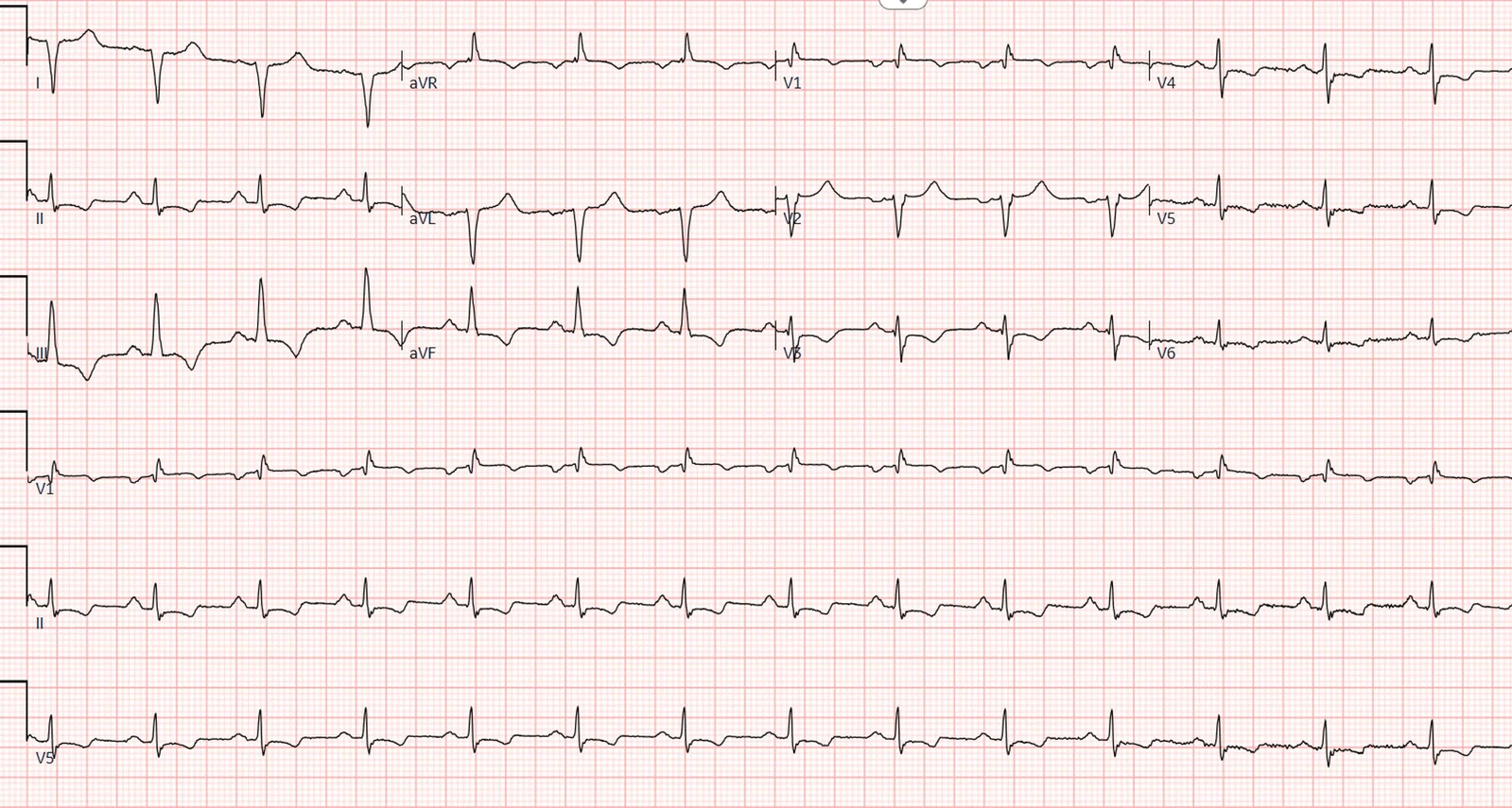
ECG demonstrating low-voltage QRS (less than 10 mm amplitude in all precordial leads) and non-specific ST-T wave changes ST-T wave pattern was unchanged from the ECG done after the prior CABG ECG: electrocardiogram; CABG: coronary artery bypass grafting

**Figure 2:**
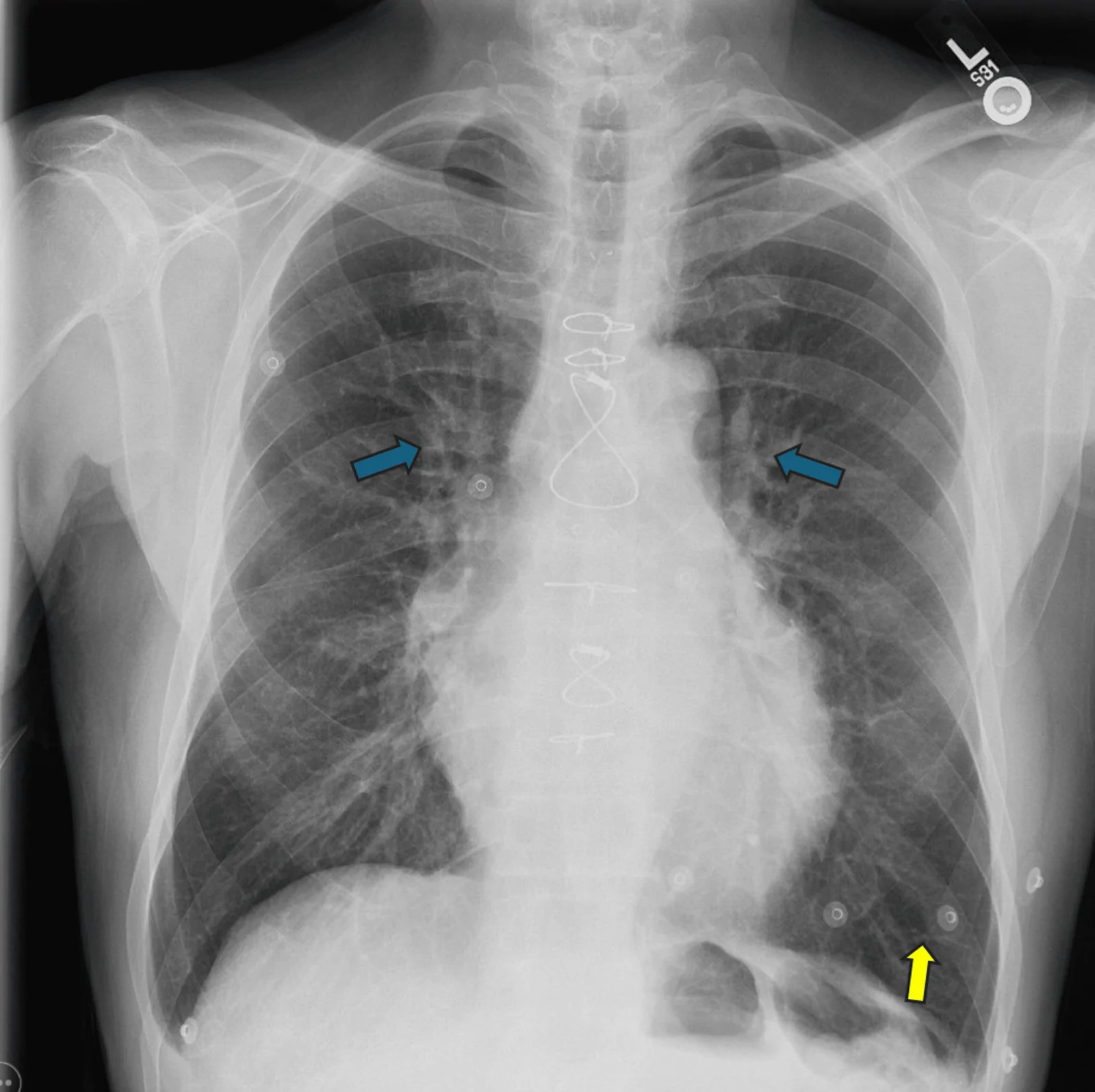
PA chest X-ray demonstrating pulmonary venous congestion with normal mediastinal width Cephalization (blue arrows). Kerley B-lines (yellow arrow) PA: posteroanterior

An echocardiogram demonstrated a large, loculated pericardial effusion located primarily posteriorly. Prominent diastolic inversion of the left ventricular inferior wall and bowing of the interventricular septum toward the right ventricle were noted. There were areas of adhesion of the cardiac surface to the pericardium (Videos 1-2, Figures 3-4). The inferior vena cava was dilated and did not demonstrate respiratory variation. There was prominent respiratory variation of the mitral inflow velocity. Although there was no collapse of the right-sided structures, these findings were consistent with cardiac tamponade. The patient underwent urgent pericardiocentesis and drain placement.

**Video 1:** Echocardiographic short-axis view The video demonstrates a large pericardial effusion with left ventricular inferior wall diastolic flattening (yellow arrow), indicative of cardiac tamponade Yellow arrow: left ventricular inferior wall. Asterisk: pericardial effusion

**Video 2:** Apical three-chamber view The video demonstrates left ventricular inferolateral wall diastolic inversion (blue arrow) and left atrial free-wall systolic inversion (red arrow). Fibrous adhesions from the pericardium to the left ventricular inferolateral wall are present (yellow arrow) Blue arrow: left ventricular inferior wall. Red arrow: left atrial free wall. Yellow arrow: fibrous adhesions. Asterisk: pericardial effusion

**Figure 3:**
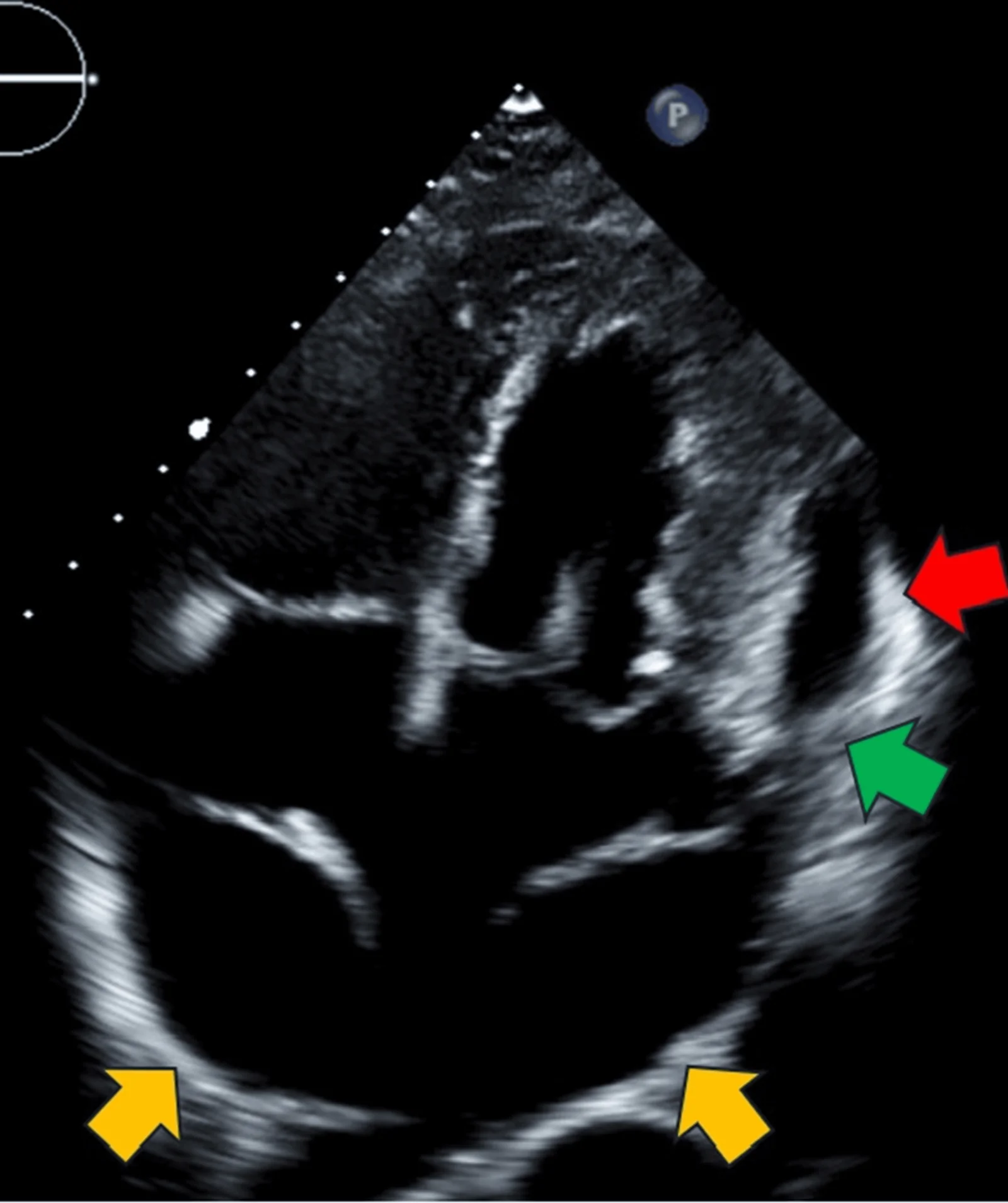
Apical four-chamber view The image shows inversion of the right and left atrial free walls, as well as pericardial effusion in the oblique sinus and in the inferolateral pericardial sac with loculation caused by fibrous adhesions (red arrow)

**Figure 4:**
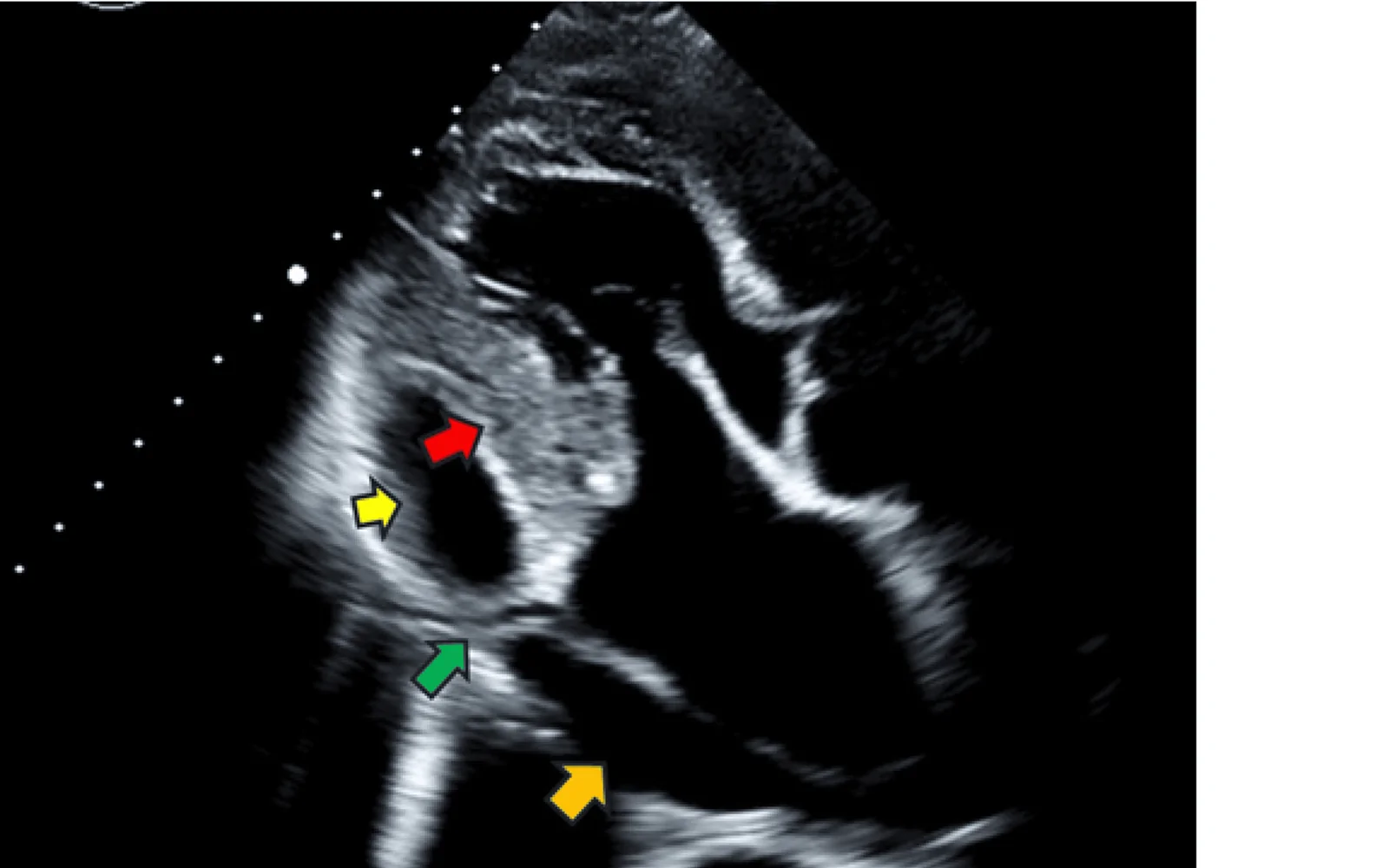
Parasternal long-axis view of the heart The figure demonstrates the loculated pericardial effusion inferolateral to the heart (yellow arrow) and in the oblique sinus (orange arrow). This is causing diastolic collapse of the left ventricular inferolateral wall and diastolic bowing of the interventricular septum towards the right ventricle (red arrows). Adhesions of the cardiac surface to the pericardium are seen (green arrow)

Pericardiocentesis initially returned 410 mL of cloudy, sanguinous fluid, which was then sent for culture and additional studies (Table 1). Given the elevated troponin levels and his prior history of CAD, the patient additionally underwent coronary angiography, demonstrating mid-LAD disease with a patent left internal mammary artery to left anterior descending graft and occlusion of the saphenous vein graft to the diagonal artery (Videos 3-5). These appeared to be chronic lesions, and angioplasty was deferred. The elevated fluid-to-serum total protein ratio (0.68) and serum-to-fluid albumin gap (0.9 g/dL) were consistent with an exudative effusion. He was treated with high-dose aspirin and colchicine for presumed idiopathic pericarditis. Fluid cytology was negative for malignancy. The drain was removed after a decrease in fluid return, and repeat echocardiography demonstrated no further pericardial effusion or left ventricular inferior wall inversion.

**Table 1:** Laboratory values of pericardial fluid and corresponding serum values NRR indicates that no reference value is available ADA: adenosine deaminase; ANA: antinuclear antibody; RBC: red blood cells; LDH: lactate dehydrogenase; ESR: erythrocyte sedimentation rate; CRP: C-reactive protein; WBC: white blood cells

Test	Value	Normal values
ADA, fluid (U/L)	6	< 40 U/L
Amylase, fluid (U/L)	34	NRR
ANA, fluid	< 1:40	NRR
RBC count (/cmm)	14,000	NRR
Total nucleated cell count (/cmm)	725	NRR
Glucose, fluid (mg/dL)	91	NRR
LDH, fluid (U/L)	224	NRR
Total protein, fluid (g/dL)	4.8	NRR
Total protein, serum (g/dL)	7.0	6.1-8.2 g/dL
Albumin, fluid (g/dL)	3.3	NRR
Albumin, serum (g/dL)	4.2	3.9-5.0 g/dL
ESR, serum (mm/hour)	0	≤ 12 mm/hour
CRP, serum (mg/dL)	0.4	< 0.8 mg/dL
WBC count, serum (x10^3^/uL)	6.4	4.16-9.95 x10^3^/uL

**Video 3:** Coronary angiography demonstrating obstructive coronary artery disease in the mid LAD and first diagonal artery Note the competitive flow of the left internal mammary artery graft in the distal LAD. Also note the presence of the pericardial drainage catheter LAD: left anterior descending artery

**Video 4:** Angiography demonstrating an occluded saphenous vein graft to the diagonal vessel Note the presence of the pericardial drainage catheter

**Video 5:** Angiography demonstrating a patent left internal mammary artery grafted to the distal LAD Note the presence of the pericardial drainage catheter LAD: left anterior descending artery

After pericardiocentesis, the patient had improvement in his symptoms and was discharged on post-procedure day four. On post-procedure day five, the pericardial fluid grew *C. acnes*, formerly known as *Propionibacterium acnes*. Blood cultures remained negative. As the patient’s effusion was exudative and the pericardial fluid was obtained under sterile conditions, the patient was diagnosed with presumed infective pericarditis. The patient was clinically well-appearing, and according to our institution’s antibiogram, *C. acnes* is recommended to be treated with penicillin or ceftriaxone. He was thus placed on a high-dose amoxicillin regimen (1000 mg TID, renally dosed) for four weeks for pericarditis, following a modified endocarditis regimen based on the POET trial [4]. Subsequent echocardiographic evaluation several weeks later demonstrated a small pericardial effusion without constrictive physiology.

## Discussion

This report featured an unusual presentation of cardiac tamponade. Isolated left ventricular chamber collapse causing cardiac tamponade is rare and most commonly seen during the perioperative phase of cardiac surgery [5]. Other rare instances of isolated left ventricular chamber collapse include regional tamponade due to a loculated pericardial effusion in the setting of malignancy. Additionally, isolated left ventricular chamber collapse can be present in cases of cardiac tamponade in patients with underlying severe pulmonary hypertension, where pericardial pressure exceeds left ventricular filling pressures, causing collapse, but does not exceed right ventricular filling pressures [6]. This case is unique in that presumed infective pericarditis led to a loculated pericardial effusion and regional tamponade as a late complication, occurring several years after cardiac surgery.

The echocardiographic findings in this case are atypical, and an understanding of the tamponade physiology is important to ensure a timely diagnosis. Cardiac tamponade occurs when the pericardial pressure exceeds the cardiac filling pressures, leading to impaired filling and chamber collapse. Because right atrial and ventricular chamber pressures tend to be lower than their left-sided counterparts, in patients with circumferential pericardial effusions, right ventricular diastolic collapse precedes left ventricular diastolic collapse. However, in this case, fibrous adhesions, likely due to a prior pericardiotomy during cardiac surgery, caused a loculated pericardial effusion. Rather than all cardiac chambers being directly affected, there was isolated left ventricular diastolic collapse without findings to suggest right ventricular diastolic collapse. This case highlights the possibility of atypical presentations of cardiac tamponade, particularly in patients with a history of prior pericardiotomy.

Infective pericarditis represents an infection of the pericardium. This may be due to direct introduction of bacteria (i.e., open heart surgery, trauma, catheter procedures), local spread, or hematogenous seeding of the pericardium [7]. Infective pericarditis may present as an acute or subacute condition and can be due to bacterial (i.e., *Staphylococcus, Streptococcus* [7], *Haemophilus* [8]), viral (i.e., Coxsackievirus B [9], cytomegalovirus [10]), fungal (Histoplasmosis [11], *Candida* [12]), or parasitic (*Toxoplasma gondii* [13]) infection.

This case presented somewhat of a diagnostic dilemma because it was unclear whether *C. acnes* from the pericardial culture represented a true infection or was the result of contamination from pericardial drain placement through the skin into the pericardial space. However, given the exudative nature of the effusion and the lack of an alternative etiology, it was treated as a true infection. *C. acnes* is an anaerobic Gram-positive bacillus that is part of the normal skin flora and is known to cause acne. Because it grows indolently and is widespread on the skin, its growth in cultures may represent a true infection or skin contamination [14]. It has previously been reported to cause infective pericarditis, although the incidence may be underreported, as it is often dismissed as a contaminant [14-16].

The patient had dental interventions performed six weeks prior to presentation. *C. acnes* is part of the normal flora of the skin and mucosal surfaces. Thus, it is possible that the patient had transient bacteremia after his dental procedure that led to infective pericarditis due to *C. acnes*. It is unlikely that the patient’s open-heart surgery several years prior led to direct inoculation of the pericardium, although it is unclear if prior instrumentation of the pericardium predisposed him to infectious pericarditis. 

If left untreated, infective pericarditis can lead to catastrophic complications. In this patient, there was echocardiographic evidence of cardiac tamponade, which can lead to hemodynamic instability. Additionally, patients may develop purulent pericarditis, in which pus is found within the pericardium [17,18]. Purulent pericarditis has a high mortality rate and is universally fatal if untreated. Long-standing infection can lead to pericardial scarring and subsequent constrictive pericarditis [19], a chronic condition that may require surgical pericardiectomy.

## Conclusions

Regional cardiac tamponade is a rare finding that is most commonly seen after cardiac surgery but can also be present in the setting of loculated pericardial effusion and severe pulmonary hypertension. Cardiologists must be aware of the possibility of atypical echocardiographic findings suggestive of cardiac tamponade, particularly isolated left ventricular collapse. Additionally, infective pericarditis is a rare condition and is diagnosed in patients with an exudative pericardial effusion and positive fluid cultures. This report describes a rare case of regional cardiac tamponade from a loculated effusion due to infective pericarditis caused by a rare organism in a patient with pericardial adhesions secondary to prior cardiac surgery performed several years earlier.
